# Supplementary material for: Ocean Acidification Amplifies the Olfactory Response to 2-Phenylethylamine: Altered Cue Reception as a Mechanistic Pathway?
Source: J Chem Ecol. 2021 May 20;47(10-11):859–76. doi: 10.1007/s10886-021-01276-9 (PMC8613125; doi:10.1007/s10886-021-01276-9)
Supplement: Supplementary file 1 — (PDF 405 KB) [file 10886_2021_1276_MOESM1_ESM.pdf]

Supplementary Information

Ocean acidification amplifies the olfactory response to 2-phenylethylamine: altered cue reception as a mechanistic pathway?

Paula Schirrmacher[1*], Christina C. Roggatz[2], David M. Benoit[3] and Jörg D. Hardege[1]

[1] Department of Biological and Marine Sciences, University of Hull, Hull, HU6 7RX, UK, E-mail: P.Schirrmacher-2018@hull.ac.uk

[2] Energy and Environment Institute, University of Hull, Hull, HU6 7RX, UK

[3] E.A. Milne Centre for Astrophysics & G.W. Gray Centre for Advanced Materials, Department of Physics and Mathematics, University of Hull, Hull, HU6 7RX, UK

1 Behaviour Data

A table of the recorded times and conditions of the behaviour experiment with hermit crabs can be found in Online Resource 1

2 Optimised Geometries of 2-Phenylethylamine

The xzy-files of the optimised geometries of 2-phenylethylamine (PEA) in gas-phase, implicit and hybrid solvation environment can be found in Online Resource 2.

3 The Effect of the Ionic Environment on PEA/PEAH$^+$ in Water

To explore the range of the dielectric constant that is relevant for hermit crab habitats, the regression equation developed by Klein and Swift (1977) was plotted for salinities and temperatures relevant for British coastal waters. Th sea temperature at the British coast varies between around 5 and 20°C (World sea temperature 2020). Inhabiting a variety of environments, *P. bernhardus* can encounter salinities from 20 ppt NaCl (Lancaster 1988), e.g. after heavy rainfall or in estuaries, to 45 ppm after evaporation in summer in tide pools. 45 ppt has been used to study the chemosensory ability of crustaceans under salinity stress (Ross and Behringer 2019). Furthermore, 20°C and 35 ppt NaCl concentration approximates the behavioural experimental condition in this study. We conclude that a dielectric constant of 80.5 to 70.2 is relevant for hermit crab habitats (see Fig. 1a).

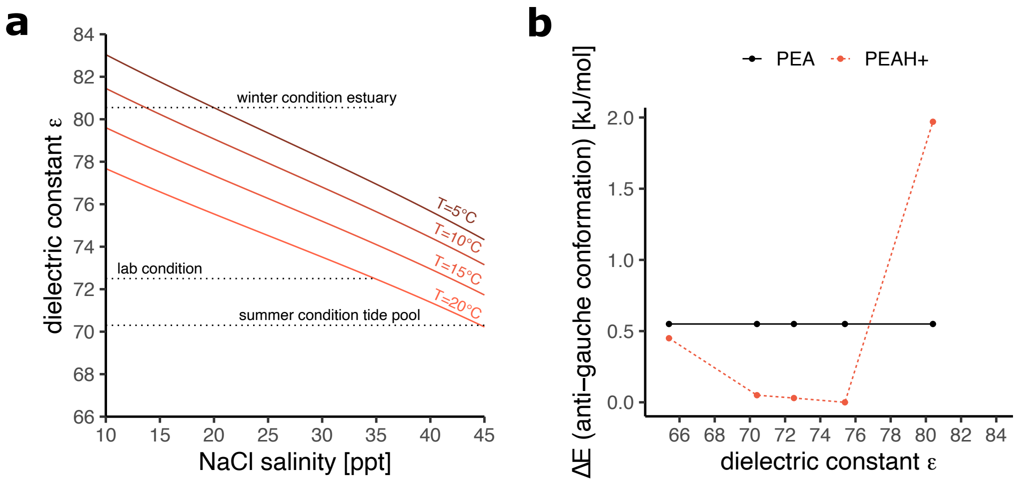

Figure 1: The dielectric constant ε of water depends on the temperature and salinity (a) and may in turn affect the energy difference between conformers (b). The first sub-figure (a) shows the dielectric constant within the salinity and temperature range relevant for hermit crab habitats. The data is fitted using the regression equation from Klein and Swift (1977). The second subfigure (b) plots the energy difference between extended (*anti*) and folded (*gauche*) PEA and $PEAH^+$ at different dielectric constants within the implicit solvation model.

Water is generally modelled implicitly using a dielectric constant of $\varepsilon = 80.4$. To explore the effect of NaCl in water on our quantum chemical models of PEA and $PEAH^+$, we conducted a set of computational experiments, whereby the dielectric constant was reduced in 3 5-point decrements, leading to a range of 80.4 - 65.4. To explicitly include the conditions during the behavioural experiments we added the dielectric constant of 72.5 to the computational set of experiments. The previously identified energetic minima of PEA and $PEAH^+$ were re-optimised in the implicit water model, applying different values for the dielectric constant. We explored the effect of the dielectric constant on the conformation, charge separation and the energetically favoured conformation of PEA and $PEAH^+$.

As Fig 1b shows, the energy difference ΔE between the energetically preferred folded and extended conformation is stable for PEA whilst it varies slightly for $PEAH^+$ at different dielectric constants. The energy difference ΔE ranges between 0 and 2 kJ/mol. However, no trend between ΔE and ε is apparent. Furthermore,

the conformation of the molecule was not affected by the decreased dielectric constant (max difference in amine side chain torsion angle $\Delta\tau = 0.16°$) and also the dipole moment was unaffected by the changes in the dielectric environment with an accuracy of $\pm 0.05\,\mathrm{D}$. We therefore conclude that the effect of the ionic environment is negligible within the accuracy of our model.

4 Computational Method Validation

To validate the quantum chemical computations, nuclear proton shieldings of the energetic minima of PEA and $PEAH^+$ were calculated in the hybrid water model (as described by Roggatz et al. (2018)). The calculated ^1H shieldings were compared to experimental shifts measured in water by plotting calculated values against experimental results and performing least-squares linear regressions for the different conformations *gauche* and *anti* of PEA/$PEAH^+$ (Fig. 2).

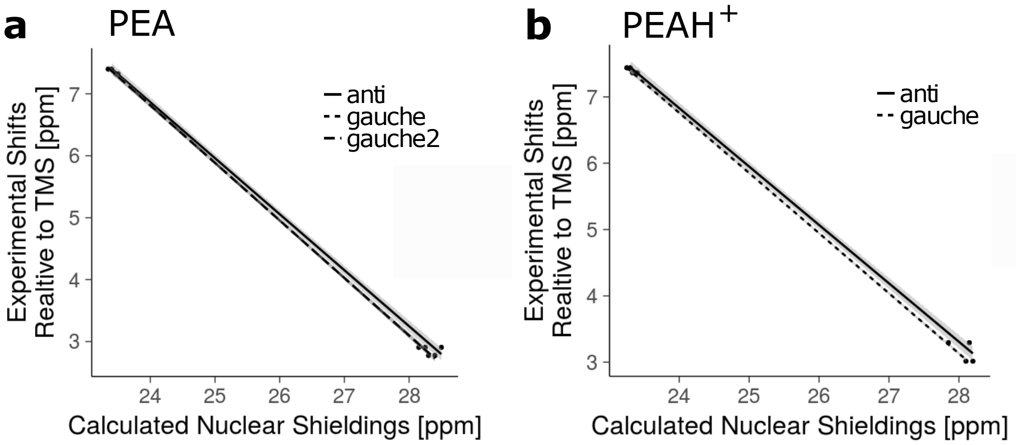

Figure 2: Calculated nuclear proton shieldings as a function of the experimental NMR ^1H shifts for the different conformations of neutral PEA (a) and $PEAH^+$ (b). The 95 % confidence interval is shown in gray. Only in the protonated state, the amino protons were measurable and thus excluded from the fit.

Thereby the calculated nuclear shieldings are absolute values whilst the experimental shifts are measured relative to the pH-insusceptible reference TMS, for which

the ^{1}H shift is set to zero. Due to the symmetry of the ring and the free rotation about single bonds (fast exchange rate) only 4 carbon-bound ^{1}H shift can be measured (Fig. 3). The corresponding calculated shieldings were averaged to facilitate comparability. As the protons bound to nitrogen were only observed in the low pH NMR experiments ($PEAH^+$) they were excluded from the linear model. H_N are known to be problematic in NMR shielding calculations as they are highly affected by hydrogen bridge networks (Frank et al. 2011).

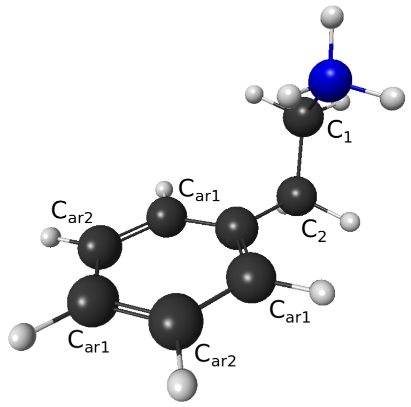

Figure 3: Structure and naming of the carbons in PEA/$PEAH^+$ to assign experimental ^{1}H NMR shifts to the calculated shieldings for both protonation states.

With the linear regression functions (eq. 1, plotted in Fig. 2) the calculated nuclear magnetic ^{1}H shieldings (σ) can be translated into chemical shifts (δ). The comparison of experimental ^{1}H shifts and calculated ^{1}H shifts is shown in table 1. It becomes apparent that the difference between folded (*gauche*) and extended (*anti*) conformation of PEA and $PEAH^+$ are reflected mainly in the chemical proton shifts of the side chain (C_1-H and C_2-H in Fig. 3) and values from the computational models of folded PEA/$PEAH^+$ are closer to the experimental results.

$$\delta = a \cdot \sigma + b \tag{1}$$

$$
\begin{aligned}
\text{for PEA :} \quad & a_{anti} = -0.90, & b_{anti} = 28.55\,\text{ppm} \\
& a_{gauche} = -0.93, & b_{gauche} = 29.12\,\text{ppm} \\
& a_{gauche2} = -0.93, & b_{gauche2} = 29.10\,\text{ppm} \\
\text{for PEAH}^+ \text{ :} \quad & a_{anti} = -0.88, & b_{anti} = 27.96\,\text{ppm} \\
& a_{gauche} = -0.91, & b_{gauche} = 28.53\,\text{ppm}
\end{aligned}
$$

Table 1: Comparison of experimental ^{1}H shifts with computational shifts of protonated and neutral PEA in water. The naming of the carbons is visualised in Fig. 3.

	neutral PEA [ppm]				$PEAH^+$ [ppm]		
	experimental	calculated			experimental	calculated	
		anti	*gauche*	*gauche 2*		*anti*	*gauche*
C_1-H	2.91	2.80	2.86	2.96	3.30	3.18	3.27
C_2-H	2.78	2.89	2.82	2.72	3.02	3.13	3.04
C_{ar1}-H	7.32	7.32	7.31	7.31	7.37	7.36	7.37
C_{ar2}-H	7.40	7.41	7.42	7.42	7.44	7.45	7.44
TMS-H	0	0.27	0.03	0.03	0	0.40	0.14

The two folded conformations (*gauche*) of neutral PEA are not separable in the linear regressions (see Fig. 2). However, the folded and extended conformation show different linear fits for both protonation states. To measure the correlation between experimental NMR shifts and calculated shieldings, the accuracy of the fit can be determined. As the calculated values are absolute but the experimental shifts are

measured relative to TMS, the calculated ^{1}H NMR shielding for TMS corresponds to the x-axis intercept. The accuracy of the fit can be estimated on the basis of the extrapolated experimental value for the calculated ^{1}H TMS shielding (Roggatz et al. 2018) (see TMS-H, last row in Table 1). Thereby, a perfect model fit would have the accuracy 0 ppm: The calculated TMS proton shielding (31.3 ppm) would coincide with the experimental NMR measurement where TMS was used as a reference (i.e. set to zero). Estimating the accuracy of the fit of the experimental findings with the computational models allows us to validate the quantum chemical calculations.

With an accuracy of ±0.03 ppm, the *gauche* conformations of neutral PEA fit the experimental values better than the *anti* conformation (accuracy ±0.27 ppm). Likewise in the protonated state, the *gauche* conformation of $PEAH^+$ reaches an accuracy of ±0.14 ppm while the accuracy of the computational model of $PEAH^+$ in the *anti* conformation is only ±0.40 ppm. For both protonation states, the folded conformation fits the experimental findings best.

Additionally, the scattering of values in the linear model can give valuable information on the goodness of fit. The 95 % confidence intervals (gray band in Fig. 2) of the linear models of the folded conformations (*gauche*) are much narrower for both protonation states, indicating a better fit of this computational model with the experimental results. The linear model of the *gauche* conformation reaches a coefficient of determination of R^2=99.99 % for protonated and R^2=99.97 % for neutral PEA, whilst the *anti* model has an R^2=99.86 % and R^2=99.89 % respectively. We conclude that PEA in the *gauche* conformation shows the best fit with the experimental data. This coincides with the conclusion based on the accuracy of the models (see above). The confidence intervals of the models are separable towards higher calculated shieldings. This is important to note as the accuracy of the fit was measured at 31.3 ppm (calculated ^{1}H TMS shielding), where the extrapolated confidence intervals are clearly separable.

In summary, ^{1}H NMR spectroscopy verifies the computational findings. A com-

parison of the accuracies as well as the linear model fits confirm that the identified global energetic minima fit best with the experimental values. The folded conformation of PEA is the energetically favoured conformation in water in both protonation states.

5 Optimised Geometries of PEA and $PEAH^+$ in TAAR1

The gaussian cube-files of the optimised geometries of PEA and $PEAH^+$ in the TAAR1 receptor pocket can be found in Online Resource 3.

6 The Effect of the Ionic Environment on Receptor-Ligand Binding

To explore the potential effect of salt and water penetration into TAAR1 on our binding models, a series of computational experiments were carried out, whereby the dielectric constant was increased from $\varepsilon = 8$ (protein background) to $\varepsilon = 10$ to include the potential effect of water and decreased to $\varepsilon = 6$ and $\varepsilon = 4$ to explore the effect of a dielectric decrement upon salt introduction. The TAAR1-PEA and TAAR1-$PEAH^+$ binding models were compared in the 4 dielectric environments by determining the distance between Asp103 oxygen atom and PEA/$PEAH^+$ nitrogen atom, the hydrogen bond angle, the torsion angle of the PEA/$PEAH^+$ amine side chain and the TAAR1-PEA/$PEAH^+$ binding energy.

The bond length between the Asp103 oxygen and PEA/$PEAH^+$ nitrogen remains at 3.10 Å and 2.52 Å respectively within all dielectric environments. The torsion angle varies slightly, by $\Delta\tau = 1.4°$ for both PEA and $PEAH^+$ without an apparent trend with the dielectric constant. The H-bond between Asp103 and the amino group of PEA/$PEAH^+$ is the same across all dielectric constants with an accuracy of

$\pm 0.1°$. Fig. 4 shows that the binding energy of PEA to TAAR1 varies only slightly with the increasing dielectric coefficient ($\Delta E = 0.6$ kJ/mol), whilst the binding energy for $PEAH^+$ with TAAR1 weakens with the increasing dielectric constant ($\Delta E = 12.1$ kJ/mol). Introducing water into TAAR1 might therefore reduce its binding affinity, whilst the addition of salt increases it.

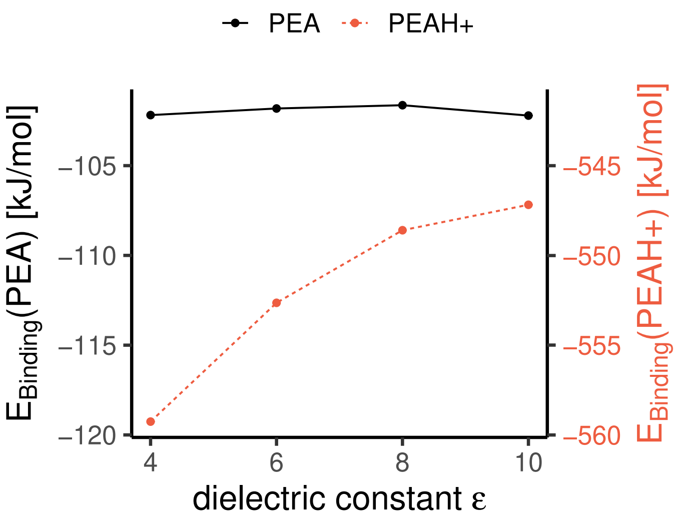

Figure 4: The binding energy of PEA/$PEAH^+$ with TAAR1 depends on the dielectric constant of the protein environment, which might be affected by water and salt ion penetration.

Keeping the perspective of this study in mind, the ratio between the binding energy of PEA and $PEAH^+$ with TAAR1 is a crucial aspect to improve our understand of pH effects on PEA-mediated behaviour. Although the binding energy of $PEAH^+$ with TAAR1 increases by 12 kJ/mol within the range of 4-10 for the dielectric constant, we still observe a five-fold increase upon protonation irrespective of the dielectric environment: The ratio of the binding energy with TAAR1 for $PEAH^+$ to PEA is 5.47 in $\varepsilon = 4$ and 5.35 in $\varepsilon = 10$.

References

Frank A, Onila I, Möller H, Exner T (2011) Toward the quantum chemical calculation of nuclear magnetic resonance chemical shifts of proteins. Proteins 79(7):2189–2202, DOI: 10.1002/prot.23041

Klein L, Swift C (1977) An improved model for the dielectric constant of sea water at microwave frequencies. IEEE Trans Antennas Propag 25(1):104–111, DOI: 10.1109/TAP.1977.1141539

Lancaster I (1988) *Pagurus bernhardus* (l.) - an introduction to the natural history of hermit crabs. Field Studies 7:189–238

Roggatz C, Lorch M, Benoit D (2018) Influence of solvent representation on nuclear shielding calculations of protonation states of small biological molecules. J Chem Theory Comput 14(5):2684–2695, DOI: 10.1021/acs.jctc.7b01020

Ross E, Behringer D (2019) Changes in temperature, pH, and salinity affect the sheltering responses of caribbean spiny lobsters to chemosensory cues. Sci Rep 9(1):1–11, DOI: 10.1038/s41598-019-40832-y

World sea temperature (2020) World sea temperatures based on daily satellite readings provided by the NOAA. URL seatemperature.org/europe/united-kingdom, visited on 10-02-2021
